# Genetic Diversity Assessed by Genotyping by Sequencing (GBS) in Watermelon Germplasm

**DOI:** 10.3390/genes10100822

**Published:** 2019-10-18

**Authors:** Kyung Jun Lee, Jung-Ro Lee, Raveendar Sebastin, Myoung-Jae Shin, Seong-Hoon Kim, Gyu-Taek Cho, Do Yoon Hyun

**Affiliations:** National Agrobiodiversity Center, National Institute of Agricultural Sciences (NAS), RDA, Jeonju 54874, Korea; lkj5214@korea.kr (K.J.L.); jrmail@korea.kr (J.-R.L.); raveendars@gmail.com (R.S.); smj1204@korea.kr (M.-J.S.); shkim0819@korea.kr (S.-H.K.); gtcho@korea.kr (G.-T.C.)

**Keywords:** Watermelon, genetic diversity, population structure, genotyping by sequencing, GBS

## Abstract

Watermelon is an economically important vegetable fruit worldwide. The objective of this study was to conduct a genetic diversity of 68 watermelon accessions using single nucleotide polymorphisms (SNPs). Genotyping by sequencing (GBS) was used to discover SNPs and assess genetic diversity and population structure using STRUCTURE and discriminant analysis of principal components (DAPC) in watermelon accessions. Two groups of watermelons were used: 1) highly utilized 41 watermelon accessions at the National Agrobiodiversity Center (NAC) at the Rural Development Administration in South Korea; and 2) 27 Korean commercial watermelons. Results revealed the presence of four clusters within the populations differentiated principally based on seed companies. In addition, there was higher genetic differentiation among commercial watermelons of each company. It is hypothesized that the results obtained from this study would contribute towards the expansion of this crop as well as providing data about genetic diversity, which would be useful for the preservation of genetic resources or for future breeding programs.

## 1. Introduction

Watermelon is an important vegetable fruit crop for human consumption. Watermelon, ranking among the top five most-frequently purchased fruits, is cultivated globally, with a per capita annual consumption of ~7 kg [1]. According to the Food and Agricultural Organization of the United Nations [2], global watermelon production was 595,422 tons in 2017 and has increased steadily, over the years. South Korea is the 19th largest watermelon producing country in the world. Watermelon is an economically important horticultural crop in South Korea, after pepper, oriental cabbage, radish, and onion [3]. 

As watermelon contains various functional factors such as lycopene and citrulline, many seed companies have been leading the development of various watermelon cultivars to cater to customers [4]. In general, the breeding system of seed companies is a method of continuous back-crossing after crossing for excellent material [5]. It is reported that the watermelon breeding system of seed companies led to gene loss in their breeding materials [4]. Guo et al. reported that there was only one SNP per 1,430 bp of watermelon cultivars between cv. Charleston Gray of the USA and cv. 97103 of China [6]. Frankel mentioned that the need for various plant genetic resources will increase in the future for the further development of scientific and technical possibilities [7]. Therefore, the results of unabated gene erosion should be reversed through all the possible means. Urgent action is needed to collect and preserve irreplaceable genetic resources.

Several molecular markers have been effectively used to assess the genetic diversity of watermelon. Isozymes [8], RAPD [9], AFLP [10], and SSR [11,12] have been used to estimate the genetic relationship among cultivated watermelons and *Citrullus* species. These studies revealed low levels of DNA polymorphism among cultivated watermelons but high genetic diversity among the *Citrullus* subspecies [12,13].

Plant genetic resources have been an intriguing research topic as one of the most essential natural resources, resulting in major advance in the field [14]. Gene banks are concerned with the maintenance of crop resources and genetic variations; recently, plant genetic resource conservation has started to gather immense attention [14,15]. In order to establish effective and efficient conservation practices for plant genetic resources, understanding the genetic diversity between and within the population is important [16]. At present, about 1100 watermelon accessions have been collected worldwide at the National Agrobiodiversity Center (NAC) at the Rural Development Administration in South Korea. However, analysis of genetic diversity in the preserved watermelon accessions in NAC is lacking. Therefore, it is necessary to learn the genetic relationship between the watermelon accessions for the efficient management of watermelon germplasms. In this study, highly utilized 41 watermelon accessions, which they have been ordered over five times from seed companies or institutes, conserved at the NAC and 27 Korean commercial watermelons were analyzed using genotyping-by-sequencing (GBS) to evaluate the genetic diversity and determine the appropriate panel of watermelon germplasm for watermelon improvement and conservation.

## 2. Materials and Methods 

### 2.1. Plant Materials

In this study, 68 watermelon accessions were used. Among them, highly utilized 41 watermelon accessions were obtained from the National Agrobiodiversity Center (NAC) at the Rural Development Administration in South Korea and 27 Korean commercial watermelons were obtained from each seed company (Table S1).

### 2.2. DNA Extraction

DNA was extracted from 30 mg of freeze-dried leaf tissue of each genotype. The QIAGEN plant mini kit (Qiagen, Valencia, CA, USA) was used for the extraction. DNA quality and quantity of each sample were determined with electrophoresis in 1% (*w/v*) agarose gels and spectrophotometry. 

### 2.3. Preparation of Genotyping-by-Sequencing Libraries

The amount of DNA was quantified using the standard procedure of Quant-iT PicoGreen dsDNA Assay Kit (Molecular Probes, Eugene, OR, USA) with Synergy HTX Multi-Mode Reader (Biotek, Winooski, VT, USA) and normalized to 12.5 ng/μL. DNA was digested with ApeKI (New England Biolabs) at 75 °C for 3 h. 

The libraries from restriction enzyme digestions for genotyping-by-sequencing (GBS) were constructed according to the protocols as described previously with minor modifications [17,18]. The restriction digestion of DNA was followed by ligation with adapters. The adapters included different barcode-containing adapters for tagging individual samples and common adapters. The ligation was performed using T4 DNA ligase (New England Biolabs) at 22 °C for 2 h and the ligase was inactivated by holding at 65 °C for 20 min.

Adapter ligated samples were pooled as one sample, and purified using NucleoSpin® Gel and PCR Clean-up Kit (MACHEREY-NAGEL GmbH & Co. KG). The pooled ligations were amplified in 50 uL reaction by multiplexing PCR using AccuPower Pfu PCR Premix (Bioneer) and 25 pmol of each primer as mentioned below: 5′- AATGATACGGCGACCACCGAGATCTACACTCTTTCCCTACACGACGCTCTTCCGATCT-3′ and 5′- CAAGCAGAAGACGGCATACGAGATCGGTCTCGGCATTCCTGCTGAACCGCTCTTCCGATCT-3′. 

The PCR products were evaluated for the distribution of fragment sizes with BioAnalyzer 2100 (Agilent Technologies). The GBS libraries were sequenced on the Illumina NextSeq500 (Illumina, San Diego, CA, USA) with the length of 150 bp single-end reads. 

### 2.4. Sequence Preprocessing and SNP Calling

The raw data file was generated in binary base call (BCL) format and directly forwarded to bcl2fastq in BaseSpace (https://basespace.illumina.com). The demultiplexing was firstly done by bcl2fastq software with one mismatch per index from provided index sequences in sample sheet. Preprocessed sequence reads were subjected to Stacks v2.0, ‘process_radtags’ module to confirm the demultiplexed reads and check restriction enzyme site. Then, a quality control for per-base quality of reads and removal of potential adaptor sequences was performed using FastQC and Cutadapt (Figure S1). Sequence preprocessing with Stacks, FastQC and Cutadapt software parameters are in Table S2 [19,20,21]. Reads were then mapped to *Citrullus lanatus* subsp. vulgaris cv. 97103 reference genome (watermelon_v1) using Bowtie2 [6,22]. Command-line Picard tools (https://broadinstitute.github.io/picard/) were used to add read groups to the reads, making available to utilize reads for Genome analysis toolkit 3.7 (GATK) pipeline. GATK was used to perform local realignments of reads to correct misalignments due to the presence of indels (“RealignerTargetCreator” and “IndelRealigner” arguments) [23]. The “HaplotypeCaller” and “SelectVariants” arguments were used for calling candidate SNPs aligned to watermelon_v1 reference genome. After raw variants were obtained, variants were filtered with “filterVariant” module in GATK to filter out according to quality score (The Phred scaled probability that a reference allele/alternative allele observed in a sample polymorphism exists, QUAL < 30), quality depth (The QUAL score normalized by allele depth for a variant, QD < 5), Fisher score (Fisher’s Exact Test to determine if there is strand bias between forward and reverse strands for the reference or alternate allele, FS >200) and with vcftools v. 0.1.15 to restrict the missing rate (--max-missing 0.95), minor allele frequency (--maf 0.05), a number of alleles (--min-alleles 2, --max-alleles 2), and mean read depth for a SNP locus (--min-meanDP 5) [23].

### 2.5. Population Structure and Genetic Diversity

The population structure was analyzed by a DAPC [24] using the *adegenet* package [25] for R software. The *find.clusters* function was used to detect the number of clusters in the population. It uses K-means clustering which decomposes the total variance of a variable into between-group and within-group components. The best number of subpopulations has the lowest associated Bayesian Information Criterion (BIC). A cross-validation function (*Xval. dapc*) was used to confirm the correct number of PC to be retained. In this analysis, the data is divided into two sets: a training set (90% of the data) and a validation set (10% of the data). The member of each group is selected by stratified random sampling, which ensures that at least one member of each group or population in the original data is represented in both training and validation sets. DAPC is carried out on the training set with variable numbers of retained PCs. The degree to which the analysis is able to predict accurately the group membership of excluded individuals (those in the validation set) is used to identify the optimal number of PCs to be retained. At each level of PC retention, the sampling and DAPC procedures are repeated many times [26]. The best number of PCs that should be retained is associated with the lowest root mean square error. The resultant clusters were plotted in a scatterplot of the first and second linear discriminants of DAPC. 

Bayesian-based clustering was performed using STRUCTURE v.2.3.4 [27] by testing three independent runs with K from 1 to 15, each run with a burn-in period of 50,000 iterations and 500,000 Markov Chain Monte Carlo (MCMC) iterations by assuming the admixture model. The output was subsequently visualized by STRUCTURE HARVESTER v.0.9.94 [28] and the most likely number of clusters was inferred according to Cattell [29]. A membership coefficient q > 0.8 was used to assign samples to clusters. Samples within a cluster with membership coefficients ≤0.8 were considered ‘genetically admixed’.

The phylogenetic network was calculated using neighbor-net in SplitsTree4 [30]. The analysis of molecular variance (AMOVA), the coefficient of genetic differentiation among populations (Fst) was estimated with the Weir and Cockerman approach using four-way comparisons of the cultivar clusters in the program vcftools v. 0.1.15 [23] and a subpopulation inbreeding coefficient (Fis) were calculated using GenAlEx software (6.5 version) [31]. Expected and observed heterozygosity (He, Ho) and the percentage of polymorphic loci were calculated using GenAlEx software.

## 3. Results

### 3.1. GBS Analysis

For the 68 watermelon accessions, sequencing of the GBS library yielded 171 million reads of good quality (Figure S1). The range of read number was varied from 1,076,854 (WM35) to 3,775,195 (WM25) with an average of 2,520,034 (Tables S3 and S4). Each of 68 sample reads was mapped to ‘*Citrullus lanatus* subsp. vulgaris cv. 97103 v1′. In the 68 watermelon accessions, an average of 2,024,970 (80.4%) reads was aligned to the reference genome. Among them, WM48 had the highest mapping rate (83.1%) and WM51 had the lowest (74.7%). 

Considering only the successfully mapped reads from 68 watermelon accessions, SNPs were discovered and genotypes were called by analyzing the single master alignment file with GATK [23]. A total of 14,052 GBS SNPs were identified and a total of 12,282 GBS SNPs were called after filtering out duplicated reads. Among them, 1770 SNPs with <5% missing data were selected, finally. The numbers of homozygote SNP loci and heterozygote SNP showed the range from 1148 (WM11) to 1715 (WM45) with an average of 1542 and 50 (WM45) to 616 (WM11) with an average of 221, respectively (Table S5). The average homozygote rate was approximately 87.4%, and the average heterozygote rate was 12.6%. 

### 3.2. Genetic Diversity of 68 Watermelon Accessions

**Figure 1:**
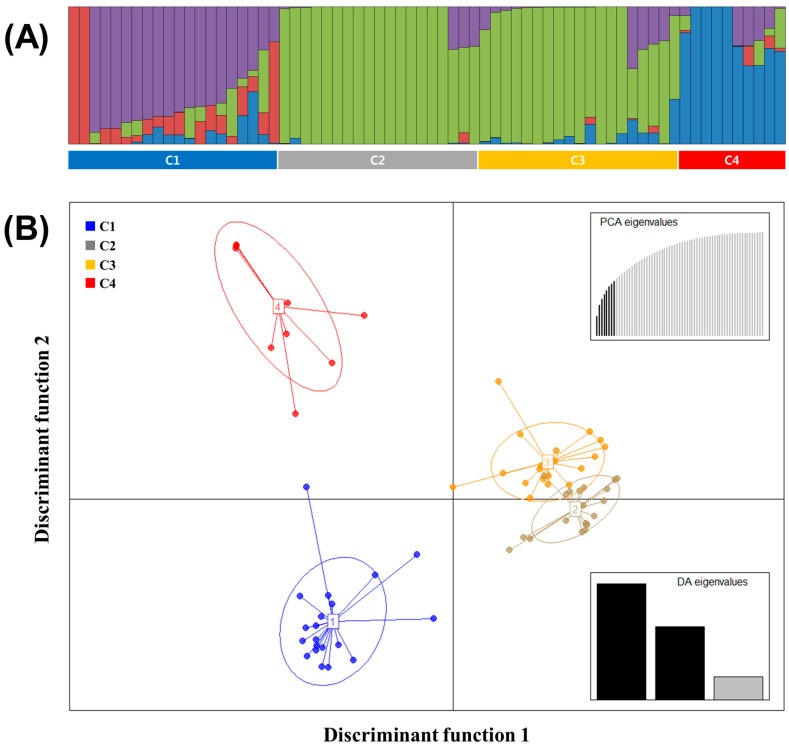
(**A**) Population structure analysis of 68 watermelon populations inferred using STRUCTURE software based on 1,770 SNPs for delta *K* = 4. (**B**) Discriminant analysis of principal components (DAPC) for 68 watermelon accessions using 1770 SNPs set. Eight PCs and three discriminant eigenvalues were retained during analyses, to describe the relationship between the clusters. The axes represent the first two Linear Discriminants (LD). Each circle represents a cluster and each dot represents an individual. Numbers represent the different subpopulations identified by DAPC analysis.

To understand the pattern of the genetic structure, a Bayesian clustering analysis in STRUCTURE and complementary ordination analysis by Discriminant Analysis of Principal Components (DAPC) were performed. The STRUCTURE results suggested the best grouping number (*K* = 4) based on the delta K (Figure S2A). Population 1, 2, 3, and 4 consisted of 5, 2, 30, and 6 accessions, respectively, and 25 accessions were identified in the admixed population (Figure 1A). 

The number of detected clusters was four, which was in concordance with the lowest BIC value obtained using *find.clusters* function. DAPC analysis was carried out using the detected number of clusters (Figure 1B). Eight first PCs (53% of variance conserved) of PCA and three discriminant eigenvalues were retained. These values were confirmed by cross-validation analysis. Cluster 1, 2, 3, and 4 consisted of 20, 19, 19, and 10 accessions, respectively. Based on the result of STRUCTURE, population 2 and 4 were identified to be present in cluster 1 and population 1 and 3 in cluster 4 and 3, respectively. Among the four clusters, cluster 1 had the highest admixed individuals (12 accessions).

**Figure 2:**
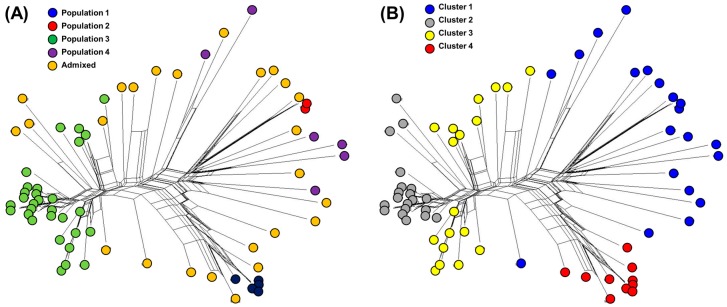
Phylogenetic network analysis calculated for 68 watermelon accessions using the neighbor-net method in Splits tree4 and compared with STRUCTURE (**A**) and DAPC (**B**).

The genetic diversity among 68 watermelon accessions was also assessed with the phylogenetic network (PN) using the neighbor-net method (Figure 2 and Table S6). The clustering of accessions in the PN was generally in agreement with STRUCTURE (Figure 2A) and DAPC (Figure 2B). The inferred sub-populations were relatively high but not completely separated. For further genetic analysis, the clusters of DAPC were used because it separated the 68 watermelon accessions than the populations of STRUCTURE in more detail. 

Results in Table 1 show that the genetic variability within clusters (93%) was greater than the variability among clusters (7%), which means that the population is genetically diverse. Allelic frequencies of clusters detected by DAPC were low differentiated heterozygotes (*Fst* = 0.068). Observed heterozygote of four clusters ranged from 0.099 (cluster 1) to 0.193 (cluster 4), with an average of 0.134 (Table 2). The range of expected heterozygote among four clusters was from 0.183 (cluster 4) to 0.342 (cluster 3), with an average of 0.293. Fixation index of four clusters was in the range of −0.039 (cluster 4) to 0.689 (cluster 1), with an average of 0.540. The percentage of polymorphic loci among the four clusters ranged from 57.5 (cluster 4) to 98.3% (cluster 3), with an average of 87.7%. 

The coefficient of genetic differentiation (*Fst*) among the four clusters was higher (0.190) in clusters 1 and 4 compared to other clusters (Table 3). Clusters with paired cluster 4 exhibited higher *Fst* value than other pairs, while lower genetic differentiation (*Fst* = 0.157) was observed between clusters 2 and 3.

### 3.3. Genetic Diversity of 27 Korean Commercial Watermelons

**Figure 3:**
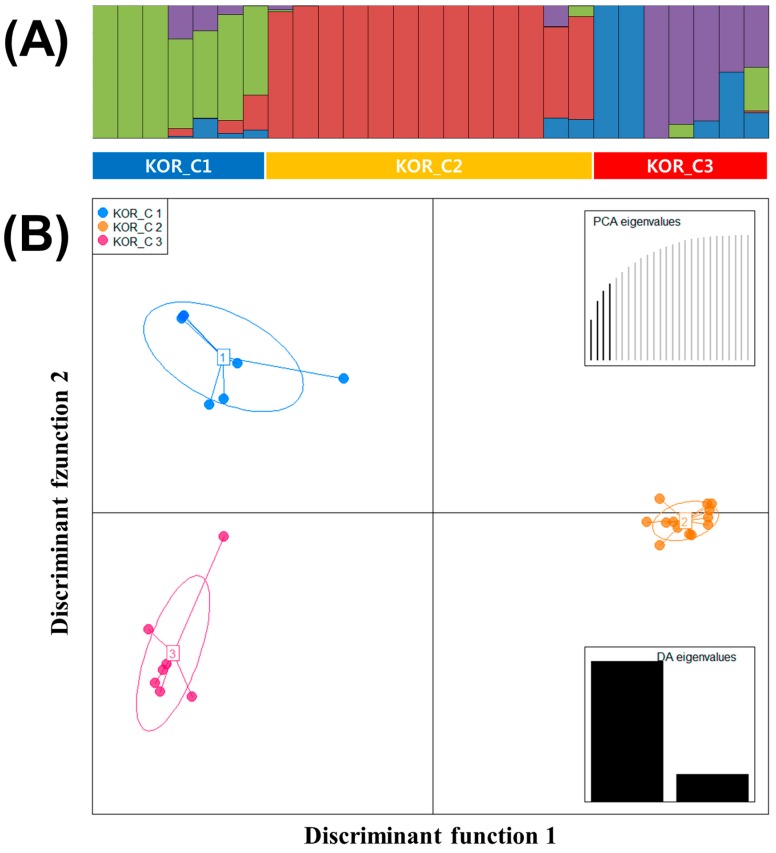
(**A**) Population structure analysis of 27 Korean commercial watermelon populations inferred using STRUCTURE software based on 1770 SNPs for delta *K* = 4. (**B**) Discriminant analysis of principal components (DAPC) for 27 Korean commercial watermelon accessions using 1770 SNPs set. Four PCs and two discriminant eigenvalues were retained during analyses, to describe the relationship between the clusters. The axes represent the first two Linear Discriminants (LD). Each circle represents a cluster and each dot represents an individual. Numbers represent the different subpopulations identified by DAPC analysis.

The STRUCTURE results of 27 Korean commercial watermelons were similar to all other watermelon accessions, the best grouping number (*K* = 4) based on the delta K (Figure S2B). Population (KOR_Pop) 1, 2, 3, and 4 consisted of 3, 11, 3, and 2 accessions, respectively, and eight accessions were in the admixed population (Figure 3A). 

Using the lowest BIC value, three clusters (KOR_C) were detected in 27 Korean commercial watermelons and these clusters were used to analyze the DAPC (Figure 3B). Four first PCs (61% of variance conserved) of PCA and two discriminant eigenvalues were preserved. In Figure 3B, Linear Discriminant 1 (LD1) separated the two major seed companies, although Linear Discriminant 2 (LD2) did not show clear criterions to separate the three clusters. KOR_C1, C2, and C3 consisted of seven, thirteen, and seven watermelon accessions, respectively. Compared to the result of STRUCTURE, KOR_Pop 1 and four admixed accessions were in KOR_1, KOR_Pop2 and two admixed accessions were in KOR_C2, and KOR_Pop3, 4 and two admixed accessions were in KOR_C3.

**Figure 4:**
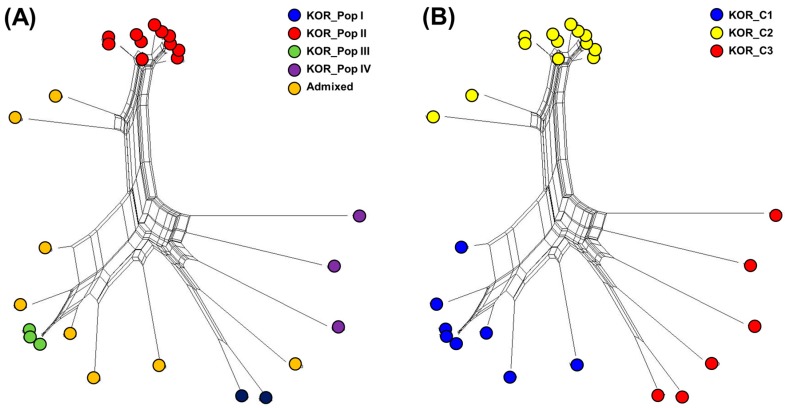
Phylogenetic network analysis calculated for 27 Korean commercial watermelon accessions using the neighbor-net method in Splits tree4 and compared with STRUCTURE (**A**) and DAPC (**B**).

The genetic diversity among 27 Korean commercial watermelon accessions was also assessed with the phylogenetic network (PN) using the neighbor-net method (Figure 4 and Table S7). Groups in the PN were, in general, in agreement with STRUCTURE (Figure 4A) and DAPC (Figure 4B), although some individuals were assigned to different clusters depending on the approach. For further genetic analysis, the clusters of DAPC were used because it discriminated the 27 Korean commercial watermelon accessions according to the seed companies.

As per the results of AMOVA (Table 4), the genetic variability within clusters (55%) was greater than the variability among the clusters (45%), signifying genetic diversity of the population. Allelic frequencies of clusters detected by DAPC were highly differentiated heterozygotes (*Fst* = 0.445). Observed heterozygote of three clusters ranged from 0.049 (KOR_C3) to 0.170 (KOR_C2) with an average of 0.094 (Table 5). The range of expected heterozygote among the three clusters was from 0.127 (KOR_C1) to 0.291 (KOR_C3) with an average of 0.199. Fixation index of four clusters was in the range of 0.144 (KOR_C2) to 0.824 (KOR_C3) with an average of 0.524. The percentage of polymorphic loci among the four clusters ranged from 45.6 (KOR_C1) to 76.7% (KOR_C3) with an average of 59.2%. 

The coefficient of genetic differentiation (Fst) among the four clusters was higher (0.424) in KOR_C1 and C2 compared to others (KOR_C1 and C3, 0.341; KOR_C2 and C3, 0.356) (Table 6). 

## 4. Discussion

### 4.1. Genotyping by Sequencing

Genotyping by sequencing (GBS) is a high-throughput and cost-effective technology to detect and genotype a large number of polymorphisms at the genome-scale [32]. Various challenges posed by complex crop genomes can be overcome by GBS [1]. Previous researches developed 1073 SNPs from 130 watermelon accessions, 5254 SNPs from 183 watermelon accessions, and 2670 SNPs from 20 watermelon accessions, respectively, for genetic mapping, SNP marker set for marker-assisted breeding, and genetic diversity studies in watermelon [1,5,33]. Guo et al. performed re-sequencing in 20 watermelon accessions including sweet, semi-wild, and wild watermelons to identify 6,784,860 candidate SNPs and 965,006 small insertions/deletions [6]. Although there is a lack of analysis on the limits of the number of watermelon accessions and the association between results of GBS and phenotypic characteristics, genetic diversity and population structure of 68 watermelon accessions have been explained using a robust set of 1770 SNPs obtained from GBS in this study. In particular, the genetic diversity of commercial watermelon accessions in Korea has been analyzed for the first time using GBS.

### 4.2. DAPC Analysis 

In this study, 68 watermelon accessions were divided into four clusters and two populations by STRUCTURE, respectively (Figure 1). In addition, 27 Korean commercial watermelon accessions were clustered based on the breeding program of seed companies in the result of DAPC, while STRUCTURE results showed two populations (Figure 3). DAPC analysis divided the population into well-defined clusters, which were related to their genetic structure, associated with provenance, ploidy, taxonomy and breeding program of the genotypes [34]. The DAPC method provides an interesting alternative to STRUCTURE software as it does not require the populations to be in HW equilibrium and can handle large sets of data without using parallel processing software [35]. However, as for other multivariate analysis, the reduction in genetic information to inter-individual or inter-population distances may represent a substantial loss of information [36]. In this study, DAPC analysis provided a more detailed clustering within watermelon accessions according to the breeding program of seed companies as compared to STRUCTURE analysis. Previous studies reported that DAPC analysis showed a more detailed clustering within landraces and bred cultivars of *Prunus avium* L. compared to STRUCTURE using SNP chips and SSR markers [36,37].

### 4.3. Genetic Diversity of 68 Watermelon Accessions

In this study, genetic differentiation (Fst) of four clusters in 68 watermelon accessions was low (*Fst* = 0.068) (Table 2). According to Wright, Fst values ranging from 0 to 0.05 indicate low, 0.05–0.15 moderate, 0.15–0.25 high, and above 0.25 very high genetic differentiations [38]. The low genetic differentiation could be attributed to the high level of gene flow among germplasm. In this study, gene flow (Nm) among 68 watermelon accessions was 4.43, suggesting a high genetic introgression. Previous studies reported similar results with a low genetic differentiation value and high gene flow among watermelon [39,40]. Gene flow <1 is considered to be low whereas Nm = 1 is considered to be moderate [41]. Moderate or relatively low levels of gene flow can significantly reduce the loss of genetic diversity [42,43]. In general, the high level of gene flow may be attributed to an exchange of genetic materials between leading to low levels of genetic differentiation [4]. In addition, high gene flow could be attributed to a high degree of movement of germplasm probably through frequent seed exchange. This practice results in low genetic variability among individuals within populations [44]. Similarly, 41 watermelon accessions among 68 accessions in this study have been distributed more than five times. Although the user’s information cannot be conclusive, it is likely that the companies have used the 41 watermelon accessions in duplicate, signifying that the same watermelon accessions were used in the development of company-specific cultivars and this may be the reason for the higher gene flow of 68 watermelon accessions. Contrary to the results of 68 watermelon accessions, the Fst (0.374) was relatively high, and the gene flow was low (0.418) among 27 Korean commercial watermelons. It appears that Korean commercial watermelons had little or no genetic exchange among seed companies.

### 4.4. Genetic Diversity of Korea Commercial Watermelon Accessions

According to the result of the DAPC analysis, 27 Korean commercial watermelons were separated by each company. Among them, 14 watermelon accessions of KOR_C1 and KOR_C3 developed in Farm-hannong and 13 watermelon accessions belonging to KOR_C2 were mixed with the three main companies, Nongwoo, Sinnong and PPS. PPS is the subsidiary company of Nongwoo and the owner of Sinnong is a former watermelon breeder of Nongwoo. It can be attributed to the same gene pool for watermelons developed or sold by the three companies. Unlike the Nongwoo (KOR_C2), the watermelon accessions by Farm-hannong were divided into two groups (KOR_C1 and C3) probably due to mergers and acquisitions between companies. The watermelons belonging to KOR_C1 were developed at Heungnongjongmyo. Heungnongjongmyo was acquired and annexed by Seminnis in 1998 and the Seminnis were annexed by Monsanto in 2005. With the acquisition of the Monsanto Korean back in 2012, the Heungnongjongmyo and the Farm-hannong became one company. Therefore, it is possible to assume that the watermelon accessions present currently in the same company are genetically different. 

## 5. Conclusions

In this study, highly utilized 41 watermelon accessions collected and conserved at the National Agrobiodiversity Center (NAC) at the Rural Development Administration in Korea and 27 commercial watermelons in Korea were analyzed using GBS to analyze the genetic diversity and population structure. The results of genetic diversity and population structure in 68 watermelon accessions showed the high level of heterozygosity in each watermelon accession and the low level of genetic differentiation between the clusters. In addition, 27 Korean commercial watermelons were divided into three clusters based on their seed companies and showed lower level of heterozygosity than 68 watermelon accessions. In general, commercial watermelons have been produced and sold by seed companies in the form of F1 [3,4]. To develop new products in seed companies, the producers focus on consumer-friendly merchantability rather than product diversity, which results in lower genetic diversity due to the limitation of their genetic resource [4,44]. If genetic diversity is low during the breeding of crops, the crop can become vulnerable to environmental changes and diseases [14]. In NAC, 1162 watermelon accessions were collected and conserved, which are mostly open-pollinated lines. In addition, the number of watermelon accessions utilized for breeding was very low compared to the number of accessions available because of their seed conditions such as low germination rate or seed volume. Levi et al. mentioned that the assembly and conservation of genetically and morphologically diverse watermelon germplasm are essential activities to ensure the current and future success of watermelon breeding programs [45]. Watermelon germplasm collections rich in genetic and phenotypic diversity are maintained in USDA/ARS, Turkey, China, and Southern Africa [45]. However, Solmaz et al. reported that most of the watermelon accessions collected in Turkey, including open-pollinated and F1 hybrid cultivars, share a similar genetic background [46]. To increase the utilization of watermelon accessions in NAC, accurate evaluation of genetic diversity and phenotypic traits is necessitated.

## Figures and Tables

**Table 1:** Results of analysis of molecular variance (AMOVA) and F-statistics for the 68 watermelon accessions.

SV ^1^	df	SS	MS	Est. Var.	%	*F*-statistics	Nm
Among clusters	3	2943.641	981.214	20.880	7%	*Fst* = 0.068	3.43
Within clusters	132	37595.411	284.814	284.814	93%		
Total	135	40539.051		305.693	100%		

^1^ SV, Source of variation; df, degrees of freedom; SS, sum of squares; MS, Mean square; Est. Var., Estimated variance; %, Percentage of variation, Nm, gene flow.

**Table 2:** Statistics of genetic variation for the 68 watermelon population.

Pop	N ^1^	Ho	He	F	%P
1	20	0.099 ± 0.004	0.323 ± 0.003	0.689 ± 0.009	96.5
2	19	0.107 ± 0.003	0.323 ± 0.003	0.658 ± 0.009	97.9
3	19	0.136 ± 0.004	0.342 ± 0.003	0.615 ± 0.009	98.9
4	10	0.193 ± 0.005	0.183 ± 0.004	-0.039 ± 0.008	57.5
Total	68	0.134 ± 0.002	0.293 ± 0.002	0.540 ± 0.005	87.7

^1^ N, Number of individuals; Ho, Observed heterozygosity; He, Expected heterozygosity; F, Fixation index; %P, Percentage of polymorphic loci.

**Table 3:** Pairwise genetic differentiation values (*Fst*) clusters of 68 watermelon accessions.

Cluster	Cluster	*Fst*	Nm
1	2	0.262	0.705
1	3	0.229	0.844
2	3	0.157	1.344
1	4	0.247	0.761
2	4	0.384	0.401
3	4	0.322	0.527

**Table 4:** Results of analysis of molecular variance (AMOVA) and F-statistics for 27 Korean commercial watermelon accessions.

SV ^1^	df	SS	MS	Est. Var.	%	*F*-statistics	Nm
Among clusters	2	5474.485	2737.243	149.115	45%	*Fst* = 0.374	0.418
Within clusters	51	9471.330	185.712	185.712	55%		
Total	53	14945.815		334.828	100%		

^1^ SV, Source of variation; df, degrees of freedom; SS, sum of squares; MS, Mean square; Est. Var., Estimated variance; %, Percentage of variation.

**Table 5:** Statistics of genetic variation for three watermelon population of 27 Korean commercial watermelon accessions.

	N ^1^	Ho	He	F	%P
KOR_C1	7	0.061 ± 0.004	0.127 ± 0.004	0.479 ± 0.014	45.6
KOR_C2	13	0.170 ± 0.006	0.181 ± 0.005	0.144 ± 0.012	55.2
KOR_C3	7	0.049 ± 0.004	0.291 ± 0.004	0.824 ± 0.010	76.7
Total	27	0.094 ± 0.003	0.199 ± 0.003	0.524 ± 0.008	59.2

^1^ N, Number of individuals; Ho, Observed heterozygosity; He, Expected heterozygosity; F, Fixation index; %P, Percentage of polymorphic loci.

**Table 6:** Pairwise genetic differentiation values (*Fst*) clusters of 27 Korean commercial watermelons.

Cluster	Cluster	Fst	Nm
1	2	0.424	0.334
1	3	0.341	0.483
2	3	0.356	0.452

## Supplementary Materials

The following are available online at https://www.mdpi.com/2073-4425/10/10/822/s1, Table S1. List of 68 watermelon accessions; Table S2. Workflow and utilized codes of sequenc preprocessinng and SNP calling; Table S3. Number of retained reads after demultiplexing using STACKS; Table S4. Number of retained reads after adaptor trimming using Cutadapt; Table S5. Information about SNPs based on GBS in watermelon accessions; Table S6. Distance matrix of 68 watermelon accessions; Table S7. Distance matrix of 27 Korean watermelon accessions; Figure S1. Sequencing quality control. (A) raw reads, (B) filtered reads; Figure S2. Relationship between delta K and K as revealed by STRUCTURE harvester. Estimation of the number of subgroups for the K values ranging from 1 to 15, by delta K values. (A) 68 watermelon accessions, (B) 27 Korean commercial watermelons.
