# Supplementary material for: Genetic Diversity Assessed by Genotyping by Sequencing (GBS) in Watermelon Germplasm
Source: Genes (Basel). 2019 Oct 18;10(10):822. doi: 10.3390/genes10100822 (PMC6826620; doi:10.3390/genes10100822)
Supplement: Supplementary file 1 [file genes-10-00822-s001.zip › Supplementary Figures.docx]

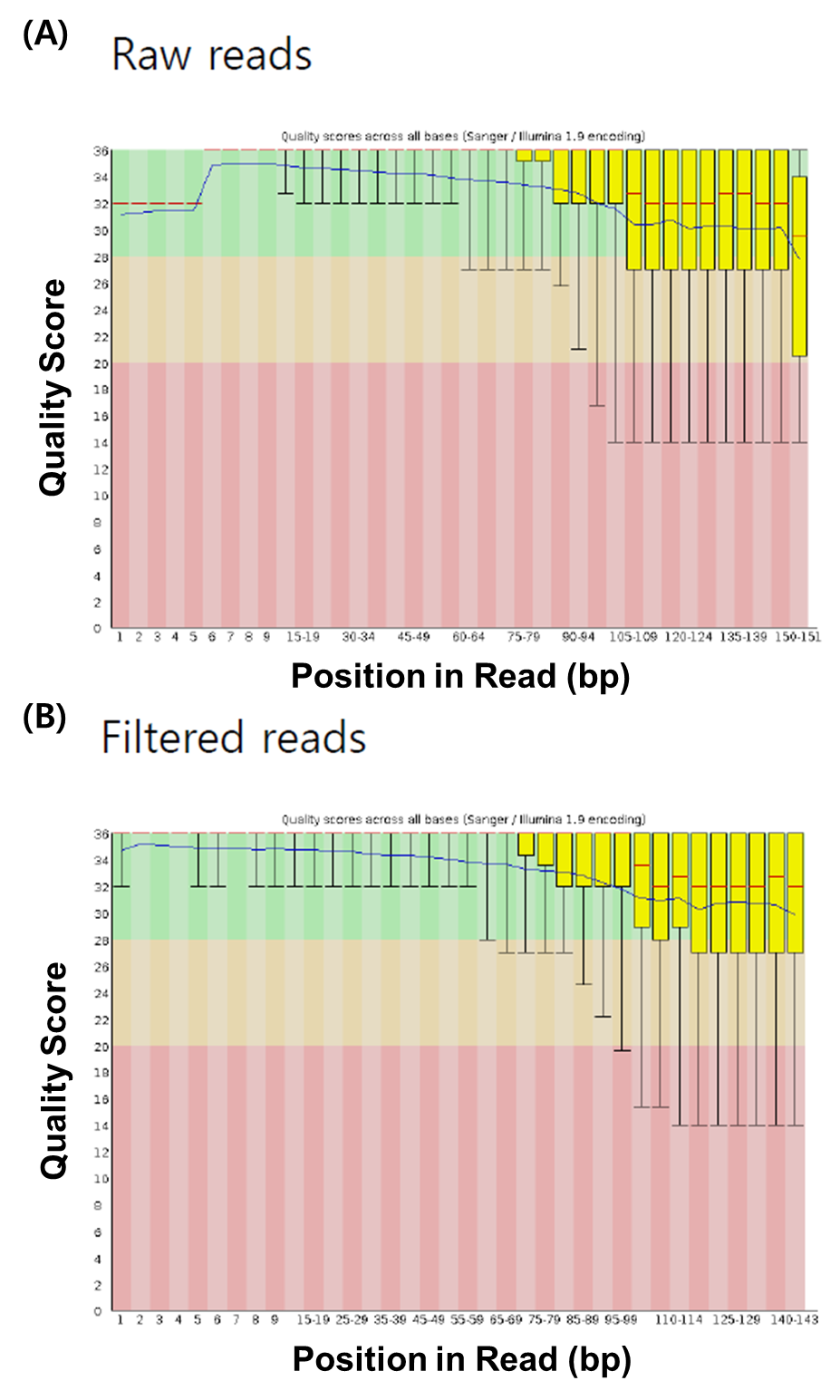


**Figure S1**. Sequencing quality control. (A) raw reads, (B) filtered reads


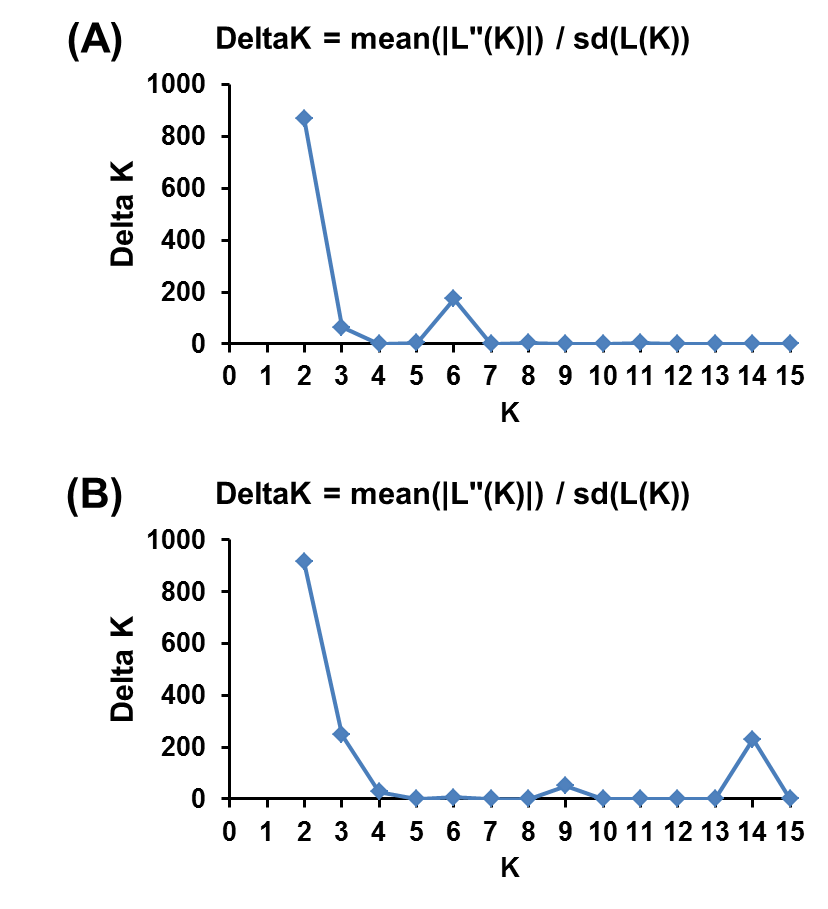


**Figure S2**. Relationship between delta K and K as revealed by STRUCTURE harvester. Estimation of the number of subgroups for the K values ranging from 1 to 15, by delta K values. (A) 68 watermelon accessions, (B) 27 Korean commercial watermelons.
